# Triacylglycerol‐Based Insulin Resistance Indices and Post‐Transplantation Diabetes Mellitus After Liver Transplantation

**DOI:** 10.1002/lipd.70048

**Published:** 2026-03-06

**Authors:** Mateo Chvatal‐Medina, Yakun Li, Adrian Post, Margery A. Connelly, Han Moshage, Stephan J. L. Bakker, Vincent E. de Meijer, Hans Blokzijl, Robin P. F. Dullaart, TransplantLines investigators

**Affiliations:** ^1^ Department of Gastroenterology and Hepatology University Medical Center Groningen, University of Groningen Groningen the Netherlands; ^2^ Department of Internal Medicine, Division of Nephrology University Medical Center Groningen, University of Groningen Groningen the Netherlands; ^3^ Labcorp Morrisville North Carolina USA; ^4^ Department of Surgery, Division of Hepato‐Pancreato‐Biliary Surgery and Liver Transplantation University Medical Center Groningen, University of Groningen Groningen the Netherlands; ^5^ Department of Internal Medicine, Division of Endocrinology University Medical Center Groningen, University of Groningen Groningen the Netherlands

**Keywords:** liver transplantation, post‐transplant diabetes mellitus, triacylglycerol/HDL‐cholesterol ratio, triacylglycerol‐glucose index

## Abstract

*Background* : Post‐transplant diabetes mellitus (PTDM) is a common complication following liver transplant (LT) and is associated with adverse outcomes. The triacylglycerol‐glucose product (TyG) and the triacylglycerol‐to‐high‐density lipoprotein (HDL) cholesterol ratio (TAG/HDL‐c) are indices that can serve as triacylglycerol‐based proxies for insulin resistance. Their relation to PTDM in liver‐transplant recipients (LTRs) is unclear. *Methods* : TyG and TAG/HDL‐c (nuclear magnetic resonance spectroscopy) were compared between LTRs without diabetes at baseline and participants without diabetes from the community‐dwelling PREVEND cohort. Among LTRs, associations with incident PTDM were determined using Cox regression analysis and logistic regression analysis with adjustment for relevant variables. *Results* : TyG was higher in 246 LTRs without diabetes (mean age 52.6 years; 53.7% male) compared to 4533 PREVEND participants without diabetes (mean 53.1 years; 48.9% male (*p* < 0.001), and confirmed using propensity score matching (*p* < 0.001). Over a median follow‐up of 7.1 (IQR 6.2–7.8) years, 31 LTRs developed PTDM (12.6% cumulative incidence). A higher baseline TyG index was associated with an increased risk of PTDM (HR per 1‐SD increase: 1.67, 95% CI 1.28–2.30; highest vs. lowest tertile HR: 1.77, 95% CI 1.14–2.64). The TAG/HDL‐c ratio followed the same trend (HR per 1‐SD increase: 1.56, 95% CI 1.14–2.15; highest vs. lowest tertile: HR 3.80, 95% CI 1.73–10.0). Findings remained in adjusted analyses and were directionally similar in logistic regression models. *Conclusions* : In LTRs, both the TyG index and the TAG/HDL‐c ratio are associated with PTDM. These indices could serve as a basis for post‐transplant risk stratification in diabetes development.

**Abbreviations**
ALT: alanine aminotransferase
AUC‐ROC: area under the curve for a receiver operating characteristic
BMI: body mass index
BP: blood pressure
CI: confidence interval
CIF: cumulative incidence function
CKD: chronic kidney disease
CNI: calcineurin inhibitors
CVD: cardiovascular disease
DBP: diastolic blood pressure
DM: diabetes mellitus
EDTA: ethylenediaminetetraacetic acid
eGFR: estimated glomerular filtration rate
HbA1c: glycated hemoglobin
HDL‐c: high‐density lipoprotein cholesterol
HOMA‐IR: homeostasis model assignment of insulin resistance
HR: hazard ratio
HTN med: antihypertensive medication
IQR: interquartile range
IRB: institutional review board
LDL‐c: low‐density lipoprotein cholesterol
Ln: natural logarithm
LP4: lipoprotein profile algorithm 4
LT: liver transplantation
LTR: liver transplant recipients
mTOR: mechanistic target of rapamycin
NFAT: nuclear factor of activated T‐cells
NMR: nuclear magnetic resonance
OR: odds ratio
PREVEND: Prevention of REnal and Vascular ENd‐stage Disease study
PTDM: post‐transplant diabetes mellitus
SBP: systolic blood pressure
SD: standard deviation
STROBE: Strengthening the Reporting of Observational Studies in Epidemiology
T1, T2, T3: tertile 1, 2, and 3
T2D: type 2 diabetes mellitus
TAG: triacylglycerol
TAG/HDL‐c: triacylglycerol‐to‐high‐density lipoprotein cholesterol ratio
TC: total cholesterol
TyG: triacylglycerol‐glucose index
UMCG: University Medical Center Groningen
VLDL: very low‐density lipoprotein

## 1 Introduction

Liver transplantation (LT) has become an effective and definitive therapy for patients with end‐stage liver disease. Given the improvement in perioperative management, long‐term survival has increased substantially since the beginning of the century (Schoening et al. 2013). However, long‐term complications contribute considerably to morbidity and mortality after LT and are becoming increasingly prevalent (Agostini et al. 2023; Craig and Heller 2021). Post‐transplant diabetes mellitus (PTDM) is one of the most common metabolic complications after LT (Lawendy et al. 2021; Wallia et al. 2016; Younossi et al. 2015). It is defined as the development of post‐transplant diabetes in patients who did not have diabetes before and is associated with graft rejection, infections, chronic kidney disease, and cardiovascular disease, which in turn may increase mortality risk in LT (Baid et al. 2001; Li et al. 2015; Lieber et al. 2019; Lv et al. 2015; Moon et al. 2006; Peláez‐Jaramillo et al. 2018).

Multiple risk factors have been identified that increase the risk of PTDM after LT, such as high body mass index (BMI), impaired fasting glucose, and specific immunosuppressive regimens such as calcineurin inhibitors (CNI) and glucocorticoids (Bai et al. 2024; Li et al. 2015; Song et al. 2016). As in type 2 diabetes mellitus (T2D), risk factors for PTDM include obesity, metabolic syndrome, insulin resistance, and beta cell dysfunction, all of which lower the threshold for post‐transplant hyperglycemia (Ducloux and Courivaud 2022; Pham et al. 2011; Zhang et al. 2023). Additionally, immunosuppressive regimens including corticosteroids, CNI, and mechanistic target of rapamycin (mTOR) inhibitors impair glucose metabolism and insulin signaling through a plethora of mechanisms, including peripheral glucose utilization, insulin secretion, and cell surface GLUT4 availability (Cai et al. 2016; Fonseca et al. 2018; Kuo et al. 2015; Li and Cummins 2022; Pereira et al. 2014).

Although there is some evidence on potential predictors of PTDM such as pre‐LT fasting glucose, age, and immunosuppressants, there are currently no reliable markers for early risk stratification reported in liver transplant recipients (LTR) that can be easily used in everyday clinical practice (Bai et al. 2024; Bhat et al. 2018; Loosen et al. 2023; Shimada et al. 2024; Vaughn et al. 2015). Both the triacylglycerol‐glucose (TyG) index and the triacylglycerol‐to‐HDL‐c (TAG/HDL‐c) ratio have emerged as triacylglycerol‐based proxies for insulin resistance, incident T2D, and cardiovascular events in the general population, as well as predictors for PTDM in kidney and heart transplant recipients (Chen et al. 2022; Kurniawan 2024; Lee et al. 2024a, b; Liang et al. 2025; Nayak et al. 2024; Peled et al. 2021). Whether the TyG index and TAG/HDL‐c ratio are associated with PTDM in LTRs remains unknown.

The present study was initiated to compare these indices between LTRs and the general population, and to investigate their associations with PTDM in LTRs.

## 2 Materials and Methods

### 2.1 Study Population and Design

To evaluate differences in TyG and TAG/HDL‐c between LTRs and the general population, and to investigate their associations with PTDM in LTRs, we conducted a cohort study using data from PREVEND and TransplantLines. The study was conducted in accordance with the STROBE (Strengthening the Reporting of Observational Studies in Epidemiology) guidelines. The PREVEND study is a population‐based cohort study that began in 1997 in the city of Groningen, the Netherlands, to investigate the role of urinary albumin excretion in the development of cardiovascular and kidney disease. It includes data on multiple health parameters related to cardiovascular, metabolic, and renal disorders. During 1997 and 1998, 85,421 people aged 28–75 were invited to participate in the study, asked for a morning urine sample, and completed a questionnaire for demographic and clinical information on cardiovascular history. In total, 40,856 individuals responded and provided a urine sample. Of these, those with urinary albumin concentrations ≥ 10 mg/L (*n* = 3395) were invited to participate in a screening visit conducted in the University Medical Center Groningen (UMCG). Exclusion criteria included pregnancy, type 1 diabetes mellitus, and insulin‐treated T2D. The screening was eventually exceeded and completed with 8592 individuals, who were included in follow‐up. Between 2001 and 2003, a second round of screening and blood sampling was conducted, involving a total of 6894 participants. Data obtained at this time point were used for comparison with the LT cohort in the current study. Due to missing values of nuclear magnetic resonance (NMR) spectroscopy‐determined lipoprotein and triacylglycerol values, data from 4837 PREVEND participants were used for comparison, where 4543 did not have T2D at baseline, and 294 had a T2D diagnosis. The PREVEND study was approved by the Institutional Review Board (IRB of the UMCG, no. 01/139). All participants provided written informed consent.

The TransplantLines cohort is a prospective observational study conducted at the UMCG, the Netherlands (NCT03272841), which includes all types of solid organ transplants from living and deceased organ donors (Eisenga et al. 2018). Cross‐sectionally and longitudinally obtained biomaterials and data are included along with extensive demographic, clinical, physical, cognitive, and psychosocial data. Blood samples for LTRs were collected as per protocol, with a median time between LT and sample draw of 9.0 years (IQR 3.6–18.0). Data collection for LTRs extended through June 2021, whereas outcomes were ascertained through February 2025. Clinical, laboratory, and outcome data were available for 367 LTRs, 246 of whom did not have either a T2D diagnosis before LT or early PTDM (< 1 year after LT), and 121 of whom had T2D before transplant or early PTDM. Exclusion criteria included individuals with no mastery of the Dutch language, inability to comprehend the questionnaire or undergo physical tests, those who had undergone re‐transplantation, and those with missing data for either triacylglycerol, glucose, or HDL‐cholesterol. TransplantLines was approved by the IRB under METc 2014/077 and was performed in accordance with the guidelines of the Declaration of Helsinki. All participants provided written informed consent.

### 2.2 Baseline Data Collection in the PREVEND and TransplantLines Cohort Study

For the PREVEND cohort, demographic data, medical history including cardiovascular disease, diabetes (determined by fasting glucose concentrations of ≥ 126 mg/dL (7.0 mmol/L), non‐fasting plasma glucose of ≥ 200 mg/dL (11.1 mmol/L) or self‐reported diagnosis; glycated hemoglobin (HbA1c) was not available in PREVEND), kidney disease and medication use were collected through standardized questionnaires and supplemented with pharmacy‐dispensing registry, as per the original cohort design. Participants maintained their regular medication during sample collection.

Outpatient visits involved standardized clinical evaluations, questionnaires, and blood collection in accordance with the study protocol. Clinical and demographic data were obtained through interviews and verified against official records. The demographic and clinical information included comorbidities, history of cardiovascular disease, preexisting diabetes (determined by fasting glucose concentrations of ≥ 126 mg/dL (7.0 mmol/L), non‐fasting plasma glucose of ≥ 200 mg/dL (11.1 mmol/L), HbA1c > 6.5% (48 mmol/mol) or self‐reported diagnosis), medication use (glucose‐ and lipid‐lowering drugs, antihypertensive medication), and hospital admissions. BMI was calculated as weight divided by length squared. Blood pressure (BP) was measured according to international guidelines. Hypertension was defined as systolic BP ≥ 140 mmHg, diastolic BP ≥ 90 mmHg, or antihypertensive drug use. Alcohol consumption was recorded in grams per day, with one drink assumed to contain 10 g of alcohol. Estimated glomerular filtration rate (eGFR) was calculated using the creatinine‐based CKD Epidemiology Collaboration equation (Inker et al. 2021).

### 2.3 Outcome Definition

The primary endpoint was incident PTDM in LTR in the TransplantLines cohort. PTDM was defined as (i) fasting plasma glucose ≥ 126 mg/dL (7.0 mmol/L), (ii) non‐fasting glucose ≥ 200 mg/dL (11.1 mmol/L), (iii) HbA1c ≥ 6.5% (48 mmol/mol), or (iv) initiation of glucose‐lowering therapy, whichever occurred first after baseline. Data were obtained from electronic medical records and validated by treating hepatologists. PREVEND participants were followed for T2D via Dutch national registries and general practitioner records. Follow‐up accrued from baseline sampling until the earliest of T2D diagnosis, death, last clinical contact, or February 1st, 2025.

### 2.4 Laboratory Measurements

Venous blood was obtained after an overnight 10‐h fast and processed within 2 h. EDTA plasma samples were sent frozen at < −70°C to Labcorp (Morrisville, NC, USA) for analysis of total cholesterol, low‐density lipoprotein (LDL) cholesterol, high‐density lipoprotein (HDL) cholesterol, and triacylglycerol on the Vantera Clinical Analyzer using the LP4 algorithm (Huffman et al. 2022). The Triacylglycerol‐glucose index (TyG) was calculated as Ln [TAG × glucose /2]; and the TAG/HDL‐c ratio was computed by dividing fasting TAG by HDL‐c, and Ln transformed for calculations. Plasma glucose, HbA1c, and serum creatinine were analyzed with standardized laboratory measurements and quality assessment control at the Department of Laboratory Medicine of the UMCG.

### 2.5 Statistical Analysis

Analyses were performed with R (version 4.4.1, R Foundation for Statistical Computing, Vienna, Austria). A two‐sided *p*‐value < 0.05 was considered statistically significant. Variables with < 30% missingness were managed with multiple imputation by predictive mean matching for numeric and by polytomous regression for categorical data. Baseline characteristics are summarized as mean ± SD, median (IQR), or *n* (%) with group differences assessed using *t*‐tests or Mann–Whitney U tests for continuous variables and χ^2^ or Fisher's exact tests for categorical variables. A 1:1 optimal propensity‐score match was performed with individuals from the PREVEND cohort, based on age, sex, BMI, eGFR, smoking, alcohol consumption, and systolic blood pressure. Our primary analysis was the association of the indices with incident PTDM using time‐to‐event models; additional analyses including cumulative incidence curves, logistic models, AUC, and subgroup analyses were performed as supportive assessments to evaluate robustness. Associations between the TyG index and the TAG/HDL‐c ratio and incident diabetes were evaluated in time‐to‐event analyses with Cox regression analysis. Logistic regression analyses were additionally performed to evaluate cumulative incidence and assess the robustness of findings. Each marker was analyzed both in a continuous and tertile‐based scale (using the lowest tertile (T1) as reference), in three models (crude; crude plus age, sex, and BMI; crude plus eGFR, CNI or steroid use, and alcohol use). Sensitivity analyses were performed in both logistic and Cox regression models, adjusting for age, sex, BMI, eGFR, CNI or steroid use, and alcohol use. Exploratory analyses of model discrimination were done using the area under the receiver operating characteristic curve (AUC‐ROC) for logistic models and Harrell's C‐statistic for time‐to‐event models. Confidence intervals were estimated using DeLong's nonparametric method and bootstrap resampling, respectively. Effect modification by age, sex, BMI, eGFR, CNI and/or steroid use, and alcohol use, was explored with stratified models and interaction terms with likelihood ratio tests. Linearity in longitudinal analysis was confirmed by comparing spline models with linear models using the likelihood ratio test. The proportional hazards assumption, tested through Schoenfeld residuals, was not violated.

## 3 Results

### 3.1 Study Populations

We compared 246 LTRs without T2D to 4543 PREVEND participants without T2D, and 121 LTRs with T2D to 294 PREVEND participants with T2D (Table 1; Figure S1). Among those without T2D, LTRs were similar in age and sex distributions compared to PREVEND, but had higher fasting glucose, lower BMI, higher BP, lower kidney function, and LTRs had a higher TyG compared to PREVEND (8.5 vs. 8.3, *p* < 0.001), although the difference in the TAG/HDL‐c ratio was not significant (0.84 vs. 0.88, *p* = 0.192). Among those with T2D, LTRs and PREVEND participants had similar TyG (9.2 vs. 9.2, *p* = 0.961) and TAG/HDL‐c (1.55 vs. 1.48, *p* = 0.325). In both cohorts, participants without T2D were younger, had lower fasting glucose, BMI, and BP, and higher eGFR than those with T2D. Participants with T2D at baseline compared to those without T2D had significantly higher TyG index (LTRs: 9.2 vs. 8.5, *p* < 0.001; PREVEND: 9.2 vs. 8.3, *p* < 0.001) and TAG/HDL‐c ratio (LTRs: 1.55 vs. 0.84, *p* < 0.001; PREVEND: 1.48 vs. 0.88, p < 0.001).

**TABLE 1:** Baseline characteristics of participants stratified by cohort.

Variable	No T2D at baseline			T2D at baseline			T2D vs no T2D	
	LTR (*n* = 246)	PREVEND (*n* = 4543)	*p* ^a^	LTR (*n* = 121)	PREVEND (*n* = 294)	*p* ^b^	LTR *p* ^c^	PREVEND *p* ^d^
Age (years)	52.6 (15.0)	53.1 (11.9)	0.514	61.1 (11.2)	63.0 (10.0)	0.089	< 0.001	< 0.001
Sex, %			0.166			0.029	0.009	0.014
Male	53.7	48.9		68.6	56.5			
Female	46.3	51.1		31.4	43.5			
SBP (mm Hg)	131 (17)	125 (18)	< 0.001	134 (17)	137 (20)	0.150	0.089	< 0.001
DBP (mm Hg)	80 (11)	73 (9)	< 0.001	78 (12)	76 (9)	0.021	0.042	< 0.001
BMI, kg/m^**2**^	25.8 (4.4)	26.5 (4.2)	0.014	28.1 (5.3)	30.0 (5.3)	0.001	< 0.001	< 0.001
eGFR (mL/min/1.73 m^**2**^)	82.2 (24.6)	92.6 (16.8)	< 0.001	70.4 (24.7)	84.1 (19.9)	< 0.001	< 0.001	< 0.001
History CVD, %			0.963			0.388	0.028	< 0.001
No	94.7	94.4		87.6	83.7			
Yes	5.3	5.6		12.4	16.3			
Smoking, %			< 0.001			< 0.001	0.089	0.033
No	89.0	72.3		95.0	78.2			
Yes	11.0	27.7		5.0	21.8			
Alcohol, %			< 0.001			< 0.001	0.007	0.405
0	67.9	34.9		82.6	38.4			
0.1–10	20.7	25.2		13.2	25.5			
10–30	9.3	20.8		1.7	17.0			
> 30	2.0	19.2		2.5	19.0			
HTN med, %			< 0.001			< 0.001	0.001	0.134
No	60.6	74.2		42.1	70.1			
Yes	39.4	25.8		57.9	29.9			
Use of steroids (%)	114 (46.3)	—	—	52 (43.0)	—	—	0.619	—
Use of mTOR inhibitors (%)	24 (9.8)	—	—	22 (18.2)	—	—	0.034	—
Use of calcineurin inhibitors (%)	174 (70.7)	—	—	87 (71.9)	—	—	0.913	—
Use of antiproliferative drugs (%)	126 (51.2)	—	—	62 (51.2)	—	—	1.000	—
Glucose (mg/dL)	95.0 (17.0)	87.1 (11.3)	< 0.001	141.6 (52.2)	144.2 (44.0)	0.613	< 0.001	< 0.001
TC (mmol/L)	4.4 (1.1)	5.4 (1.0)	< 0.001	4.2 (0.9)	5.3 (1.2)	< 0.001	0.061	0.061
HDL‐c (mmol/L)	1.5 (0.42)	1.3 (0.30)	< 0.001	1.3 (0.46)	1.1 (0.26)	< 0.001	< 0.001	< 0.001
LDL‐c (mmol/L)	2.3 (0.79)	3.6 (0.90)	< 0.001	2.0 (0.63)	3.4 (1.0)	< 0.001	0.002	0.004
TAG (mmol/L)	1.4 (0.68)	1.3 (0.88)	0.382	1.9 (1.03)	1.8 (1.36)	0.687	< 0.001	< 0.001
TyG index	8.5 [8.1–8.8]	8.3 [8.0–8.7]	< 0.001	9.2 [8.8–9.6]	9.2 [8.8–9.6]	0.916	< 0.001	< 0.001
TAG/HDL‐c ratio	0.84 [0.57–1.33]	0.88 [0.57–1.43]	0.192	1.55 [0.87–2.14]	1.48 [1.01–2.29]	0.325	< 0.001	< 0.001

*Note:* Data are shown as mean ± SD for normally distributed variables, median [IQR] for non‐normal variables, and percentages for categorical variables.

Abbreviations: BMI, body mass index; CVD, cardiovascular disease; DBP, diastolic blood pressure; eGFR, estimated glomerular filtration rate; Glucose, fasting plasma glucose (mg/dL); HDL‐c, high‐density lipoprotein cholesterol; HTN med, antihypertensive medication; LDL‐c, low‐density lipoprotein cholesterol; LTR, liver transplant recipients; mTOR: mechanistic target of rapamycin; PREVEND, Prevention of REnal and Vascular ENd‐stage Disease study; SBP, systolic blood pressure; T2D, type 2 diabetes; TAG, triacylglycerol; TAG/HDL‐c ratio, log‐transformed triacylglycerol‐to‐HDL‐cholesterol ratio [Ln(TAG/HDL‐c)]; TC, total cholesterol; TyG index, triacylglycerol‐glucose index [Ln(TAG × Glucose/2)].

^a^ *p* Denotes the *p*‐value for the comparison of LTR versus PREVEND without T2D at baseline.

^b^ *p* Denotes the *p*‐value for the comparison of LTR versus PREVEND with T2D at baseline.

^c^ *p* Denotes the comparison of LTR with and without T2D at baseline.

^d^ *p* denotes the comparison of PREVEND with and without T2D at baseline.

To minimize confounding due to different characteristics between PREVEND and LTRs, we conducted 1:1 propensity score matching on age, sex, BMI, eGFR, smoking status, alcohol, and SBP. Matching yielded 246 pairs without T2D and 121 pairs with T2D (Table S1). In the set without T2D, LTRs retained higher fasting glucose, lower total cholesterol and LDL‐c, and higher HDL‐c compared to PREVEND participants. The TyG index was still higher in LTRs compared to PREVEND controls (8.5 vs. 8.3, *p* = 0.001), and the TAG/HDL‐c ratio remained comparable (0.84 vs. 0.89, *p* = 0.210). In the matched set with T2D, TyG (9.2 vs. 9.2, *p* = 0.653) and TAG/HDL‐c (1.55 vs. 1.43, *p* = 0.652) were also similar. In each cohort, differences persisted between those with and without T2D, both in TyG (LTRs: 9.2 vs. 8.5, *p* < 0.001; PREVEND: 9.2 vs. 8.3, *p* < 0.001) and TAG/HDL‐c (LTRs: 1.55 vs. 0.84, *p* < 0.001; PREVEND: 1.43 vs. 0.89, *p* < 0.001).

### 3.2 Time‐To‐Event Analyses: Association Between Baseline TyG Index and the TAG/HDL‐c Ratio With Post‐Transplant Diabetes Mellitus (PTDM)

**FIGURE 1:**
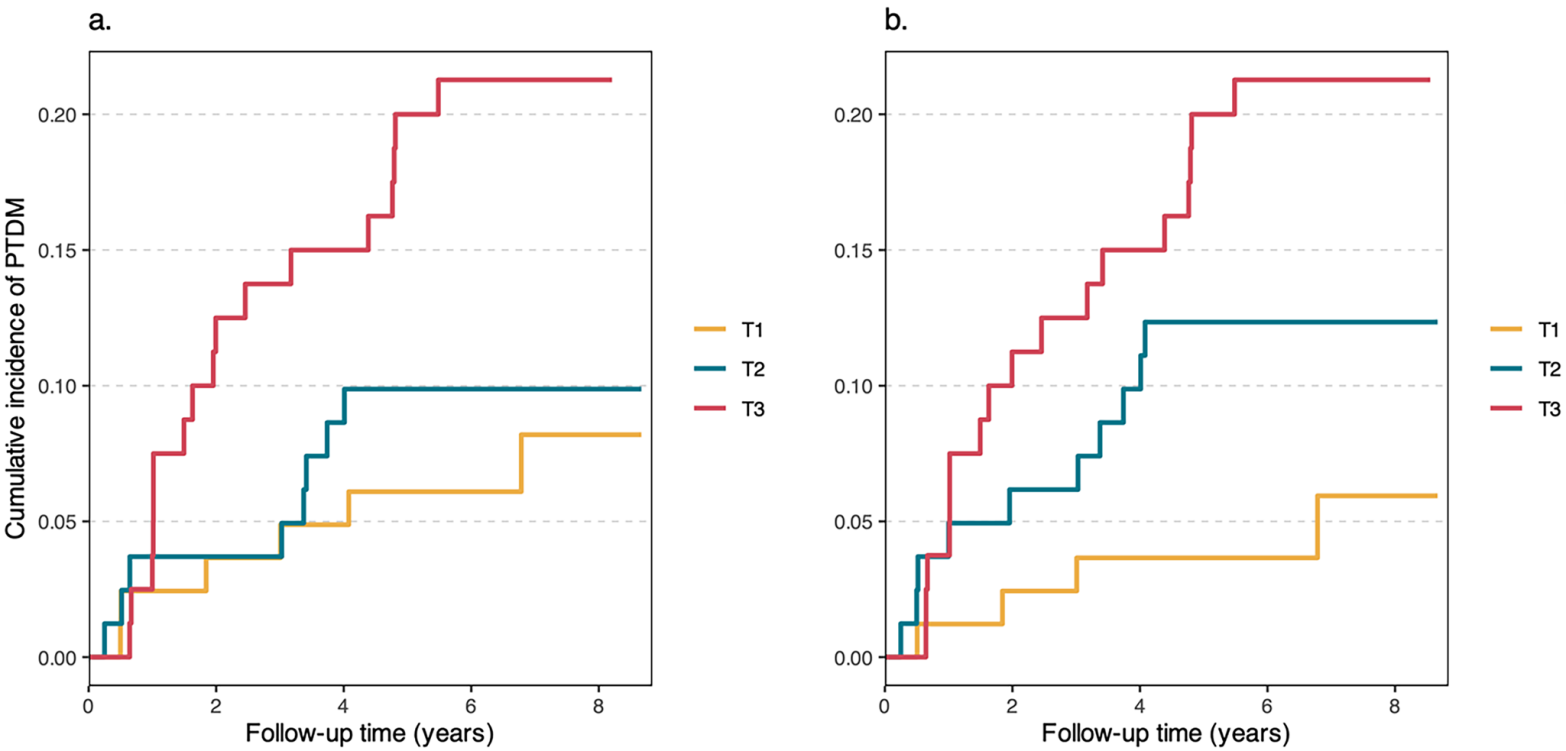
Cumulative incidence of post‐transplant diabetes mellitus by TyG index and TAG/HDL‐c ratio tertiles.

We next analyzed the association between these triacylglycerol‐based indices and PTDM in the 246 participants from the LTR cohort who did not have T2D at baseline (Figure S1). There were 31 incident cases of PTDM, with a cumulative incidence of 12.6% over a median follow‐up period of 7.1 years (IQR 6.2–7.8). LTRs were divided into tertiles based on the TyG index and TAG/HDL‐c ratio. Cumulative Incidence Function (CIF) curves showed a gradient of risk of PTDM for both indices, with the lowest risk in recipients with TyG and TAG/HDL‐c in the first tertile and the highest risk in those in the highest tertile (Figures 1; *p* = 0.021 and *p* = 0.008, respectively).

Figure 1, a Cumulative incidence function of PTDM by TyG Index tertiles, and b. Cumulative incidence function by PTDM by TAG/HDL‐c Ratio tertiles.

Cox regression models also showed that for each 1‐SD increase in the TyG index, LTRs had a 67% increase in the hazard of developing PTDM (HR 1.67, 95% CI 1.28–2.30; Table 2). The association also remained significant in the adjusted models considering (i) age, sex, and BMI (Model 2; HR 1.32, 95% CI 1.03–1.60); and kidney function, use of CNI or steroids, and alcohol use (Model 3; HR 1.49, 95% CI 1.19–1.86). For TAG/HDL‐c, each 1‐SD rise increased the hazard by 56% in unadjusted models (HR 1.56, 95% CI 1.14–2.15). The association waned after adjusting for age, sex, and BMI (HR 1.27, 95% CI 0.98–1.67), and was significant after adjusting for eGFR, use of CNI or steroids, and alcohol (HR 1.29, 95% CI 1.08–1.54). Secondary analyses adjusting for all six covariates showed similar results for each marker (TyG HR 1.26, 95% CI 1.06–1.47; TAG/HDL‐c HR 1.22, 95% CI 1.00–1.47).

**TABLE 2:** Association between the TyG index, the TAG/HDL‐c ratio, and post‐transplant diabetes mellitus by Cox regression analysis.

Model	HR [95% CI] per 1‐SD increment	T1 (Reference)	T2 HR [95% CI]	T3 HR [95% CI]
TyG index				
Model 1 (Unadjusted)	1.67 [1.28–2.30] *p* < 0.001	Reference	0.97 [0.66–1.39] *p* = 0.900	1.77 [1.14–2.64] *p* = 0.016
Model 2 (+age, sex, BMI)	1.32 [1.03–1.60] *p* = 0.024	Reference	0.97 [0.63–1.52] *p* = 0.944	1.55 [1.05–2.41] *p* = 0.040
Model 3 (+eGFR, CNI or steroids, alcohol)	1.49 [1.19–1.86] *p* < 0.001	Reference	1.06 [0.80–1.39] *p* = 0.744	1.48 [1.09–1.95] *p* = 0.008
TAG/HDL‐c ratio				
Model 1 (Unadjusted)	1.56 [1.14–2.15] *p* = 0.012	Reference	2.17 [0.95–5.33] *p* = 0.080	3.80 [1.73–10.0] *p* < 0.001
Model 2 (+age, sex, BMI)	1.27 [0.98–1.67] *p* = 0.092	Reference	1.46 [0.80–2.49] *p* = 0.284	1.92 [1.18–3.02] *p* = 0.016
Model 3 (+eGFR, CNI or steroids, alcohol)	1.29 [1.08–1.54] *p* = 0.012	Reference	1.15 [0.77–1.69] *p* = 0.520	1.78 [1.16–2.50] *p* = 0.008
Secondary analysis: All six covariates				
TyG	1.26 [1.06–1.47] *p* = 0.020	Reference	1.14 [0.72–1.73] *p* = 0.632	1.61 [1.10–2.37] *p* = 0.020
TAG/HDL‐c	1.22 [1.00–1.47] *p* = 0.068	Reference	1.09 [0.76–1.60] *p* = 0.680	1.49 [1.07–2.13] *p* = 0.032

*Note:* Significant HRs are given in bold. The TyG index and the TAG/HDL‐c ratio are Ln‐transformed. Secondary analyses include adjustment for all covariates: age, sex, BMI, eGFR, CNI or steroid use, alcohol use.

Abbreviation: HR: hazard ratio.

Furthermore, compared to those in the lowest tertile, individuals with TyG index in the highest tertile had a 1.77‐fold higher hazard of developing PTDM in crude models (95% CI 1.14–2.64); this association was attenuated but remained after adjusting for age, sex and BMI (HR 1.55, 95% CI 1.05–2.41), as well as after adjusting for eGFR, CNI or steroid use, and alcohol (HR 1.48, 95% CI 1.09–1.95). Similarly, those with the highest TAG/HDL‐c ratio had the highest risk of PTDM, which, after adjustment for age, sex and BMI, was 1.92‐fold higher compared to the lowest tertile (95% CI 1.18–3.02), and after adjustment for eGFR, CNI or steroid use, and alcohol was 1.78‐fold higher (95% CI 1.16–2.50). Secondary analyses with full adjustment also showed significance in the highest tertile (TyG HR 1.61, 95% CI 1.10–2.37; TAG/HDL‐c HR 1.49, 95% CI 1.07–2.13).

### 3.3 Association Between Baseline TyG Index and the TAG/HDL‐c Ratio With Post‐Transplant Diabetes Mellitus by Logistic Regression Analysis

In the unadjusted logistic regression analysis model, each 1‐standard deviation (SD) increment in TyG corresponded to a 74% increase in the odds of PTDM (OR 1.74, 95% CI 1.23–2.42; Table 3). After adjustment for age, sex and BMI (Model 2), the estimate attenuated but remained significant (OR 1.35, 95% CI 1.07–1.74), as well as after adjustment for eGFR, CNI or steroid use, and alcohol use (OR 1.39, 95% CI 1.15–1.71). When TyG was categorized into tertiles, the adjusted odds of PTDM in the highest tertile, compared to the lowest tertile, were 1.68 (95% CI 1.02–3.16) and 1.94 (95% CI 1.22–3.05) in Models 2 and 3, respectively. Secondary analyses adjusting for all covariates showed a significant association for both markers (TyG OR 1.32, 95% CI 1.06–1.62; TAG/HDL‐c OR 1.26, 95% CI 1.04–1.54).

**TABLE 3:** Logistic regression showing association between the TyG index and TAG/HDL‐c ratio with post‐transplant diabetes mellitus.

Model	OR [95% CI] per 1‐SD increment	T1 (Reference)	T2 OR [95% CI]	T3 OR [95% CI]
TyG				
Model 1 (Unadjusted)	1.74 [1.23–2.42] *p* < 0.001	Reference	1.08 [0.54–1.93] *p* = 0.876	2.42 [1.34–4.43] *p* = 0.008
Model 2 (+age, sex, BMI)	1.35 [1.07–1.74] *p* = 0.020	Reference	0.99 [0.49–1.84] *p* = 0.960	1.68 [1.02–3.16] *p* = 0.040
Model 3 (+eGFR, CNI or steroids, alcohol)	1.39 [1.15–1.71] *p* < 0.001	Reference	1.20 [0.77–1.92] *p* = 0.496	1.94 [1.22–3.05] *p* = 0.008
TAG/HDL‐c				
Model 1 (Unadjusted)	1.56 [1.08–2.26] *p* = 0.012	Reference	1.55 [0.84–2.76] *p* = 0.184	2.81 [1.57–4.80] *p* < 0.001
Model 2 (+age, sex, BMI)	1.30 [0.99–1.71] *p* = 0.056	Reference	1.52 [0.82–2.90] *p* = 0.204	2.19 [1.18–4.08] *p* = 0.004
Model 3 (+eGFR, CNI or steroids, alcohol)	1.38 [1.09–1.74] *p* = 0.008	Reference	1.23 [0.75–2.02] *p* = 0.460	1.97 [1.21–3.09] *p* = 0.012
Secondary Analysis: All Six Covariates				
TyG	1.32 [1.06–1.62] *p* < 0.001	Reference	1.14 [0.70–1.77] *p* = 0.608	1.65 [1.09–2.40] *p* = 0.032
TAG/HDL‐c	1.26 [1.04–1.54] *p* = 0.024	Reference	1.14 [0.71–1.84] *p* = 0.604	1.63 [1.06–2.49] *p* = 0.044

*Note:* Significant ORs are given in bold. The TyG index and the TAG/HDL‐c ratio are Ln‐transformed. Secondary analyses include adjustment for all covariates: age, sex, BMI, eGFR, CNI or steroid use, alcohol use.

Abbreviation: OR: Odds ratio.

The TAG/HDL‐c ratio showed a similar pattern, with each 1‐SD increment corresponding to a 56% increase in odds of PTDM, which waned slightly after adjustment set for age, sex, and BMI (OR 1.30, 95% CI 0.99–1.71), and was significant in the model adjusting for eGFR, CNI or steroid use, and alcohol (OR 1.38, 95% CI 1.09–1.74). In the adjusted models, those in the highest tertile of TAG/HDL‐c had a 2.19‐fold (95% CI 1.18–4.08) and 1.97‐fold (95% CI 1.21–3.09) greater odds of PTDM than those in the lowest tertile. Secondary analyses with full adjustment also showed significance in the highest tertile (TyG OR 1.65, 95% CI 1.09–2.40; TAG/HDL‐c OR 1.63, 95% CI 1.06–2.49).

### 3.4 Discrimination of TyG and TAG/HDL‐c in Predicting Post‐Transplant Diabetes Mellitus

**FIGURE 2:**
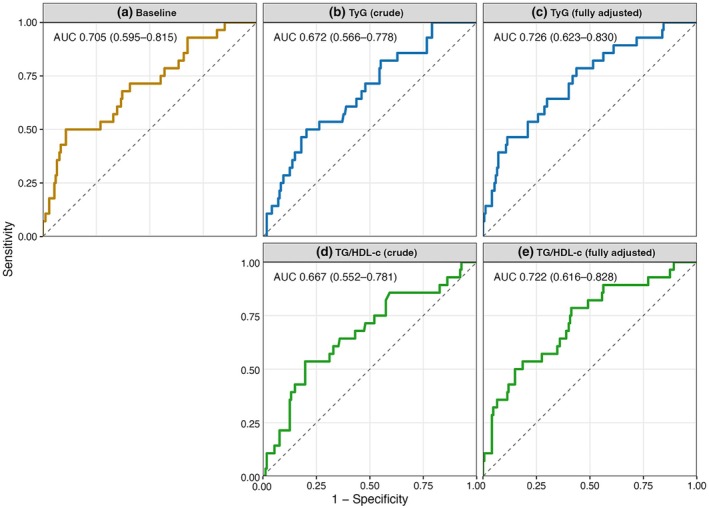
AUC‐ROC for model discrimination of TyG and TAG/HDL‐c for post‐transplant diabetes mellitus.

Exploratory analyses were conducted to assess the discriminatory ability of TyG and TAG/HDL‐c for PTDM. A baseline model which included age, sex, BMI, eGFR, CNI and/or steroids, and alcohol use yielded a C‐statistic of 0.693. When TyG and TAG/HDL‐c were evaluated individually in crude models, they showed fair performance with C‐statistics of 0.662 and 0.657, respectively. Combining each biomarker with all six clinical covariates slightly improved model discrimination to 0.713 and 0.714 for TyG and TAG/HDL‐c, respectively; however, these increments were not statistically significant compared to models only with each marker (TyG change in C‐statistics: 0.051, *p* = 0.169; TAG/HDL‐c change in C‐statistics: 0.057, *p* = 0.167). Similar findings were obtained from logistic regression models: Compared to the baseline model with only age, sex, BMI, eGFR, CNI or steroids, and alcohol use (AUC‐ROC 0.705, 95% CI 0.595–0.815), the individual markers also showed fair discrimination (TyG AUC‐ROC 0.672, 95% CI 0.566–0.778; TAG/HDL‐c AUC‐ROC 0.667, 95% CI 0.552–0.781; Figure 2). Fully adjusted models increased discrimination to 0.726 for TyG (95% CI 0.623–0.830, change in AUC: 0.056) and 0.722 for TAG/HDL‐c (95% CI 0.616–0.828, change in AUC: 0.055); however, these increments were not statistically significant (TyG *p* = 0.151, TAG/HDL‐c *p* = 0.185).

Discriminative performance of logistic regression models, with area under the ROC curve and its 95% confidence interval within each panel. A: Baseline model including age, sex, body mass index, estimated glomerular filtration rate, use of calcineurin inhibitors or steroids, and alcohol consumption. B‐C: TyG index models, crude and fully adjusted (TyG + six covariates). D‐E: TAG/HDL‐c ratio models, crude and fully adjusted (TAG/HDL‐c + six covariates).

### 3.5 Stratified Analysis on Association Between Baseline TyG and TAG/HDL‐c With Post‐Transplant Diabetes Mellitus

Exploratory stratified analyses in time‐to‐event and logistic regression models revealed apparent differences in effect sizes between men and women across age categories, with varying BMI, kidney function, use of CNI and/or steroids, and alcohol use (Figure S2). Magnitudes and direction of effect sizes were similar and consistent in both time‐to‐event and cross‐sectional analyses (Figure S1). The association of TyG appeared stronger in those aged 60 or older (HR 1.73, 95% CI 1.13–2.64), in men (HR 2.04, 95% CI 1.32–3.15), those with higher BMI (HR 1.73, 95% CI 1.15–2.60), those with lower kidney function (HR 2.45, 95% CI 1.21–2.26), those not using alcohol (HR 1.92, 95% CI 1.23–3.01), and those using CNI or steroids (HR 1.74, 95% CI 1.22–2.49; Table 4). Findings from the logistic models showed a similarly robust association. In addition, the association of PTDM with TAG/HDL‐c appeared stronger at older age (HR 1.65, 95% CI 1.07–2.53), in men (HR 1.69, 95% CI 1.09–2.60), with higher BMI (HR 1.59, 95% CI 1.07–2.37), those not using alcohol (HR 2.08, 95% CI 1.30–3.33), and those using CNI or steroids (HR 1.56, 95% CI 1.08–2.25), and logistic regression analysis corroborated these findings and also showed a significant association in lower eGFR (OR 2.54, 95% CI 1.10–7.11). Despite the apparent stronger association between strata, there was no evidence that the strength of the association varied across subgroups since none of the interaction terms were significant Table 4.

**TABLE 4:** Subgroup relative effect measures for TyG and TAG/HDL‐c in relation to post‐transplant diabetes mellitus, stratified by relevant variables.

Subgroup	Cox Regression			Logistic Regression		
	HR (95% CI)	*p*	*p* (interaction)	OR (95% CI)	*p*	*p* (interaction)
TyG index						
Age, years: ≥ 60	**1.73** **(1.13–2.64)**	**0.012**	0.406	**1.86** **(1.11–3.21)**	**0.020**	0.386
Age, years: < 60	1.26 (0.69–2.32)	0.455		1.29 (0.66–2.44)	0.438	
Sex: Male	**2.04** **(1.32–3.15)**	**0.001**	0.170	**2.33** **(1.41–4.02)**	**0.001**	0.108
Sex: Female	1.26 (0.71–2.24)	0.437		1.21 (0.64–2.21)	0.542	
BMI, kg/m^2^: ≥ 25	**1.73** **(1.15–2.60)**	**0.009**	0.612	**1.90** **(1.19–3.16)**	**0.009**	0.491
BMI, kg/m^2^: < 25	1.42 (0.74–2.72)	0.289		1.42 (0.70–2.78)	0.307	
eGFR, mL/min: ≥ 60	**1.52** **(1.02–2.26)**	**0.039**	0.254	**1.56** **(1.00–2.42)**	**0.047**	0.200
eGFR, mL/min: < 60	**2.45** **(1.21–4.94)**	**0.013**		**3.06** **(1.29–8.88)**	**0.019**	
Alcohol: Yes	1.44 (0.76–2.73)	0.267	0.462	1.42 (0.68–2.93)	0.338	0.377
Alcohol: No	**1.92** **(1.23–3.01)**	**0.004**		**2.13** **(1.25–3.78)**	**0.007**	
CNI or Steroids: Yes	**1.74** **(1.22–2.49)**	**0.002**	0.828	**1.83** **(1.23–2.78)**	**0.003**	0.830
CNI or Steroids: No	1.55 (0.60–4.02)	0.365		1.61 (0.56–5.87)	0.400	
TAG/HDL‐c ratio						
Age, years: ≥ 60	**1.66** **(1.07–2.57)**	**0.022**	0.531	**1.83** **(1.11–3.16)**	**0.023**	0.445
Age, years: < 60	1.31 (0.72–2.38)	0.377		1.33 (0.70–2.51)	0.372	
Sex: Male	**1.71** **(1.10–2.67)**	**0.018**	0.368	**1.91** **(1.16–3.24)**	**0.013**	0.253
Sex: Female	1.21 (0.64–2.30)	0.553		1.16 (0.58–2.28)	0.662	
BMI, kg/m^2^: ≥ 25	**1.60** **(1.07–2.40)**	**0.023**	0.491	**1.73** **(1.09–2.82)**	**0.022**	0.450
BMI, kg/m^2^: < 25	1.20 (0.60–2.42)	0.601		1.24 (0.58–2.59)	0.566	
eGFR, mL/min: ≥ 60	1.45 (0.96–2.18)	0.075	0.417	1.47 (0.95–2.31)	0.087	0.292
eGFR, mL/min: < 60	2.08 (0.96–4.47)	0.062		**2.54** **(1.10–7.11)**	**0.045**	
Alcohol: Yes	1.07 (0.52–2.24)	0.849	0.134	1.03 (0.44–2.19)	0.950	0.085
Alcohol: No	**2.08** **(1.30–3.33)**	**0.002**		**2.42** **(1.39–4.48)**	**0.003**	
CNI or Steroids: Yes	**1.56** **(1.08–2.25)**	**0.017**	0.671	**1.62** **(1.09–2.45)**	**0.019**	0.574
CNI or Steroids: No	2.18 (0.58–8.13)	0.247		2.51 (0.72–16.5)	0.219	

*Note:* Significant HRs and ORs are given in bold.

Abbreviations: CNI: calcineurin inhibitors, eGFR: estimated glomerular filtration rate, HR: hazard ratio, OR: odds ratio.

## 4 Discussion

This study employs two simple triacylglycerol‐derived proxies of insulin resistance to assess associations with PTDM in LTRs. The TyG index was found to be higher in LTRs without diabetes compared to PREVEND participants without diabetes, a finding that was confirmed using a propensity score matching procedure. Moreover, both the TyG index and the TAG/HDL‐c ratio were higher in LTRs with than in those without T2D at baseline, as well as in PREVEND participants with than in those without T2D.

In a prospective analysis among LTRs, both the TyG index and the TAG/HDL‐c ratio were associated with developing PTDM. Over a median follow‐up of over 7 years, around 13% of LTRs who initially did not have diabetes developed PTDM, which aligns with previous reports in PTDM and is higher than the incidence of diabetes in the general population (Lawendy et al. 2021; Sokooti et al. 2021a, b; Wallia et al. 2016; Younossi et al. 2015). Each 1‐SD higher TyG index was associated with a 33% higher adjusted hazard of PTDM when accounting for covariables like age, sex, and CMI, and those in the highest TyG tertile had over a 1.5‐fold higher risk compared to the lowest tertile. On the other hand, the TAG/HDL‐c ratio showed a consistent direction of effect despite an attenuation of the association after adjustment in the per 1‐SD models, with the tertile comparisons still showing an over 1.9‐fold higher adjusted hazard in the highest tertile relative to the lowest tertile in the model accounting for age, sex, and BMI. The logistic models corroborated these findings, both in continuous and tertile analyses. Secondary analyses were performed adjusting for age, sex, BMI, eGFR, CNI and/or steroid use, and alcohol use. The results were similar to those from other models and reinforce independent associations. However, these analyses were done exploratorily, since the number of events in this cohort limits the amount of covariates that should be included in the regression models.

Multiple human studies have tried to identify potential clinical and laboratory parameters that could serve as predictive markers for PTDM after LT. These include advanced recipient age, higher pre‐transplant BMI, and pre‐LT impaired fasting glucose (Abdelrahman et al. 2024; Bai et al. 2024). Factors such as post‐transplant weight gain, hypertriglyceridemia, hypertension, and sarcopenia have also been linked with a higher risk of developing PTDM in LTRs (Abdelrahman et al. 2024; Lee et al. 2024a, b). Other possible factors involved include viral hepatitis as the etiology of end‐stage liver disease, high donor BMI, graft steatosis, and prolonged hospital stay after LT (Abdelrahman et al. 2024; Bai et al. 2024; Sun et al. 2021). Novel biomarkers, such as elevated interleukin‐6, and the presence of genetic polymorphisms, including the PNPLA3 I148M variant (a risk factor for metabolic dysfunction‐associated steatotic liver disease), have also been associated with an increased risk for PTDM in LTRs (Abdelrahman et al. 2024). However, the level of evidence for these predictors varies, and only certain limited factors, such as older age and certain immunosuppressive regimens such as CNI and steroids, are consistent across studies; none have established proper cut‐off values for clinical practice so far. In the present study, we applied two independent adjustment sets, given the limited number of PTDM cases. The models accounted for (i) age, sex, and BMI, and for (ii) eGFR, CNI or steroid use, and alcohol use, which collectively demonstrated that even after adjustment for relevant clinical, demographic, and lifestyle covariates that would plausibly condition the onset of PTDM, the association between these variables and PTDM remained robust. Furthermore, we performed exploratory analysis for discriminative ability of these indices, and demonstrated a fair performance for both markers, especially when fitted in adjusted models for age, sex, and BMI.

The TyG index and the TAG/HDL‐c ratio are simple and pragmatic surrogates of insulin resistance that can be used to predict incident T2D and cardiometabolic events in the general population (Chen et al. 2022; da Silva et al. 2020; Dakota et al. 2025; Zhong et al. 2025). Previous work in the context of LT has shown the usefulness of the TyG index for predicting stroke and mortality, and in the context of kidney and heart transplant, it has also been proven as a valuable predictor of PTDM (Ding et al. 2024; Peled et al. 2021; Sokooti et al. 2021a, b). Nevertheless, there was no previous evidence regarding the association of the TyG or TAG/HDL‐c ratio with PTDM in LTRs. Hence, our study is the first to propose that these markers may also carry prognostic value following LT, where immunosuppression, inflammation, and changes in body composition interact to promote metabolic dysfunction (Gabrielli et al. 2024).

Our observation that both indices are independently associated with the development of PTDM in LTRs is clinically plausible. Several pathways could link an elevated TyG index and a higher TAG/HDL‐c ratio to post‐transplant hyperglycemia and PTDM. Corticosteroids potentiate hepatic gluconeogenesis and antagonize insulin signaling in muscle and adipose tissue, while promoting adipocyte lipolysis (Geer et al. 2014; Rahimi et al. 2020). The resultant rise in circulating free fatty acids enhances hepatic VLDL production and fasting hypertriglyceridemia, which increases TyG and TAG/HDL‐c (Cole et al. 1982; Geer et al. 2014; Rahimi et al. 2020). Calcineurin inhibitors such as tacrolimus and cyclosporine also disrupt glucose metabolism through beta‐cell dysfunction by inhibiting nuclear factor of activated T‐cells (NFAT), reducing insulin gene transcription, and accelerating beta‐cell apoptosis (Heit et al. 2006; Oetjen et al. 2003; Soleimanpour et al. 2010). mTOR inhibitors characteristically raise triacylglycerol and lower HDL‐c, which inflates the TAG/HDL‐c ratio (Aggarwal et al. 2006; Mulder et al. 2022). Beyond immunosuppression, post‐transplant body composition changes, including weight gain, an increase in visceral adiposity, and sarcopenia, impair insulin‐mediated glucose disposal (Cigrovski Berkovic et al. 2024; Kallwitz 2015). Additionally, these metabolic changes favor atherogenic dyslipidemia, which elevates both TyG and TAG/HDL‐c (Cigrovski Berkovic et al. 2024). In our stratified analyses, effect sizes appeared larger in men, in participants with BMI ≥ 25 kg/m^2^, and in those aged ≥ 60 years. These subgroup patterns are biologically plausible for insulin resistance phenotypes; however, we could not conclude formal effect modification due to the lack of statistically significant p‐interaction values.

To contextualize the baseline profiles of LTRs, we compared them with the population‐based PREVEND cohort. When comparing LTRs without diabetes to participants from the PREVEND cohort study without diabetes, LTRs had higher fasting glucose and HDL‐c, but similar triacylglycerol, lower total cholesterol, and LDL‐c. Upon propensity score matching on age, sex, BMI, eGFR, smoking, alcohol, and systolic blood pressure, LTRs without diabetes still showed higher fasting glucose as well as a higher TyG index compared to matched PREVEND participants, which highlights the potential relevance of this index specifically in the post‐transplant scenario.

Our findings conceivably have several implications clinically as well as for future research. First, these indices show promise for risk stratification in the pre‐transplant and early post‐transplant scenario. The TyG index and TAG/HDL‐c ratio can be easily measured in fasting plasma to determine LTRs with the highest risk for PTDM development. Second, these indices can be used for targeted monitoring and prevention because patients with the highest TyG and TAG/HDL‐c may benefit from intensified surveillance, early counseling, and caution in titration or de‐escalation of medication, as well as an early start of glucose‐lowering medication.

This study has several strengths and limitations. It is the first to evaluate the association of TyG index and TAG/HDL‐c ratio with PTDM in LTRs. It is a robust assessment of the association between the indices and clinical factors, made possible by the design of the TransplantLines cohort and the PREVEND cohort. Furthermore, the measurements of triacylglycerol and HDL‐c were made using a precise NMR spectroscopy assay. Although these lipid and lipoprotein measures can be easily and accurately assayed in the routine laboratory, this was done because routine lipid measures were not available in all patients. Moreover, this approach opens the avenue of future research into the role of lipoprotein subfractions on PTDM in LTRs (Sokooti et al. 2021a, b). Our study also has limitations. First, LTRs from the TransplantLines cohort were included at various time points after LT, which can result in disparities when comparing their baseline situation. For this reason, we conducted not only time‐to‐event analyses but also logistic regression analyses. The convergence of results from both analyses underscores the consistency of our findings. However, it is worth mentioning that given the limited number of PTDM events, secondary analyses including logistic models, as well as cumulative incidence functions, discrimination and subgroup/interaction analyses should be interpreted cautiously and be viewed as supportive. Second, plasma insulin was not measured in the LTR cohort, precluding a direct comparison of TyG and TAG/HDL‐c with insulin resistance indices such as homeostasis model assessment of insulin resistance (HOMA‐IR). Notably, the TyG index has been proposed to obviate the necessity to document insulin resistance as shown in kidney transplant recipients (Colladant et al. 2023; Sokooti et al. 2023). Third, only 31 PTDM events accrued. This limited statistical power, and heterogeneity in the original etiologies of end‐stage liver disease could not be accounted for, and were our rationale for performing predictive analysis with full adjustment sets for regression only in an exploratory fashion. We also acknowledge that the limitation on event occurrence is in part because of excluding those with early transient hyperglycaemia, which is itself a risk factor for PTDM. Though our aim was on persistent PTDM beyond the early post‐transplant period, we consider that future works on PTDM could be focused on also on patients with transient acute hyperglycaemia. Fourth, since this is an observational study, inferences about causality are precluded. Finally, given the predominant North European origin of patients in our study, these findings cannot be extrapolated per se to other ethnicities.

## 5 Conclusion

In this prospective cohort of LTRs followed for over 7 years, two simple fasting‐based indices, namely the TyG index and TAG/HDL‐c ratio, were associated with the development of PTDM. We propose that these indices could serve in post‐transplant risk stratification for PTDM in LTRs. Further validation in other LTR cohorts may underscore the validity of these indices in PTDM prediction in this patient category.

## Author Contributions

Conceptualization: Mateo Chvatal‐Medina. and Robin P.F. Dullaart. Data curation: Mateo Chvatal‐Medina and Robin P.F. Dullaart. Formal analysis: Mateo Chvatal‐Medina and Robin P.F. Dullaart. Methodology: Mateo Chvatal‐Medina, Margery A. Connelly, and Robin P.F. Dullaart. Resources, writing – original draft: Mateo Chvatal‐Medina and Robin P.F. Dullaart. Writing – review and editing: Yakun Li, Adrian Post, Margery A. Connelly, Han Moshage, Stephan J.L. Bakker, Vincent E. de Meijer, and Hans Blokzijl. All authors have substantially contributed to the manuscript design and/or revision and have approved this final version of the work. The authors have agreed to take accountability for all aspects of this study.

## Funding

The TransplantLines Biobank received funding from Astellas BV. From 1997 to 2003, the Dutch Kidney Foundation funded the infrastructure of the PREVEND program (Grant E.033). The UMCG provided infrastructure support from 2003 to 2006. Dutch Heart Foundation funded research on lipid metabolism (Grant 2001–005). Funders were not involved in any of the study phases and did not influence the decision to publish.

## Ethics Statement

The study was conducted in accordance with the Declaration of Helsinki. The PREVEND study was approved by the IRB of the UMCG (no. 01/139). The TransplantLines study was approved by the IRB of the UMCG (no. 14/077) and is registered at ClinicalTrials.gov (NCT03272841).

## Consent

All participants provided written informed consent prior to enrollment.

## Conflicts of Interest

M.A.C. is an employee of Labcorp, holds stock in Labcorp, and was involved in the NMR spectroscopy measurements. Labcorp was not involved in the study design, data management, formal analysis, or the decision to publish the results. The other authors declare no conflicts of interest.

## Supporting information


**Data S1:** lipd70048‐sup‐0001‐Supinfo.docx.

## Data Availability

The data that support the findings of this study are available on request from the corresponding author. The data are not publicly available due to privacy or ethical restrictions.

## References

[lipd70048-bib-0001] Abdelrahman, Z. , A. P. Maxwell , and A. J. McKnight . 2024. “Genetic and Epigenetic Associations With Post‐Transplant Diabetes Mellitus.” Genes 15, no. 4: 4. 10.3390/genes15040503. PMC11050138 38674437

[lipd70048-bib-0002] Aggarwal, D. , M. L. Fernandez , and G. A. Soliman . 2006. “Rapamycin, an mTOR Inhibitor, Disrupts Triglyceride Metabolism in Guinea Pigs.” Metabolism, Clinical and Experimental 55, no. 6: 794–802. 10.1016/j.metabol.2006.01.017. 16713440

[lipd70048-bib-0003] Agostini, C. , S. Buccianti , M. Risaliti , et al. 2023. “Complications in Post‐Liver Transplant Patients.” Journal of Clinical Medicine 12, no. 19: 6173. 10.3390/jcm12196173. 37834818 PMC10573382

[lipd70048-bib-0004] Bai, R. , R. An , S. Chen , et al. 2024. “Risk Factors and Prediction Score for New‐Onset Diabetes Mellitus After Liver Transplantation.” Journal of Diabetes Investigation 15, no. 8: 1105–1114. 10.1111/jdi.14204. 38641877 PMC11292396

[lipd70048-bib-0005] Baid, S. , A. B. Cosimi , M. L. Farrell , et al. 2001. “Posttransplant Diabetes Mellitus in Liver Transplant Recipients: Risk Factors, Temporal Relationship With Hepatitis C Virus Allograft Hepatitis, and Impact on Mortality.” Transplantation 72, no. 6: 1066–1072. 10.1097/00007890-200109270-00015. 11579302

[lipd70048-bib-0006] Bhat, V. , M. Tazari , K. D. Watt , and M. Bhat . 2018. “New‐Onset Diabetes and Preexisting Diabetes Are Associated With Comparable Reduction in Long‐Term Survival After Liver Transplant: A Machine Learning Approach.” Mayo Clinic Proceedings 93, no. 12: 1794–1802. 10.1016/j.mayocp.2018.06.020. 30522594

[lipd70048-bib-0007] Cai, H. , L. Q. Dong , and F. Liu . 2016. “Recent Advances in Adipose mTOR Signaling and Function: Therapeutic Prospects.” Trends in Pharmacological Sciences 37, no. 4: 303–317. 10.1016/j.tips.2015.11.011. 26700098 PMC4811695

[lipd70048-bib-0008] Chen, Y. , Z. Chang , Y. Liu , et al. 2022. “Triglyceride to High‐Density Lipoprotein Cholesterol Ratio and Cardiovascular Events in the General Population: A Systematic Review and Meta‐Analysis of Cohort Studies.” Nutrition, Metabolism, and Cardiovascular Diseases 32, no. 2: 318–329. 10.1016/j.numecd.2021.11.005. 34953633

[lipd70048-bib-0009] Cigrovski Berkovic, M. , V. Šeša , I. Balen , Q. Lai , H. Silovski , and A. Mrzljak . 2024. “Key Challenges of Post‐Liver Transplant Weight Management.” World Journal of Transplantation 14, no. 4: 95033. 10.5500/wjt.v14.i4.95033. 39697459 PMC11438933

[lipd70048-bib-0010] Cole, T. G. , H. G. Wilcox , and M. Heimberg . 1982. “Effects of Adrenalectomy and Dexamethasone on Hepatic Lipid Metabolism.” Journal of Lipid Research 23, no. 1: 81–91. 7057114

[lipd70048-bib-0011] Colladant, M. , M. Chabannes , T. Crepin , J. Bamoulid , C. Courivaud , and D. Ducloux . 2023. “Triglyceride‐Glucose Index and Cardiovascular Events in Kidney Transplant Recipients.” Kidney International Reports 8, no. 11: 2307–2314. 10.1016/j.ekir.2023.08.021. 38025208 PMC10658270

[lipd70048-bib-0012] Craig, E. V. , and M. T. Heller . 2021. “Complications of Liver Transplant.” Abdominal Radiology 46, no. 1: 43–67. 10.1007/s00261-019-02340-5. 31797026

[lipd70048-bib-0046] da Silva, A. , A. P. S. Caldas , D. M. U. P. Rocha , and J. Bressan . 2020. “Triglyceride‐Glucose Index Predicts Independently Type 2 Diabetes Mellitus Risk: A Systematic Review and Meta‐Analysis of Cohort Studies.” Primary Care Diabetes 14, no. 6: 584–593. 10.1016/j.pcd.2020.09.001. 32928692

[lipd70048-bib-0013] Dakota, I. , W. Huang , M. A. Wijayanto , et al. 2025. “Prognostic Value of Triglyceride‐Glucose Index on Predicting Major Adverse Cardiovascular Events in Hypertensive Patients: A Systematic Review and Meta‐Analysis.” American Journal of Preventive Cardiology 22: 100996. 10.1016/j.ajpc.2025.100996. 40290417 PMC12032867

[lipd70048-bib-0014] Ding, Z. , M. Ge , Y. Tan , C. Chen , and Z. Hei . 2024. “The Triglyceride‐Glucose Index: A Novel Predictor of Stroke and All‐Cause Mortality in Liver Transplantation Recipients.” Cardiovascular Diabetology 23, no. 1: 27. 10.1186/s12933-023-02113-x. 38218842 PMC10787491

[lipd70048-bib-0015] Ducloux, D. , and C. Courivaud . 2022. “Prevention of Post‐Transplant Diabetes Mellitus: Towards a Personalized Approach.” Journal of Personalized Medicine 12, no. 1: 116. 10.3390/jpm12010116. 35055431 PMC8778007

[lipd70048-bib-0016] Eisenga, M. F. , A. W. Gomes‐Neto , M. van Londen , et al. 2018. “Rationale and Design of TransplantLines: A Prospective Cohort Study and Biobank of Solid Organ Transplant Recipients.” BMJ Open 8, no. 12: e024502. 10.1136/bmjopen-2018-024502. PMC6318532 30598488

[lipd70048-bib-0017] Fonseca, A. C. R. G. , E. Carvalho , J. W. Eriksson , and M. J. Pereira . 2018. “Calcineurin Is an Important Factor Involved in Glucose Uptake in Human Adipocytes.” Molecular and Cellular Biochemistry 445, no. 1–2: 157–168. 10.1007/s11010-017-3261-0. 29380240 PMC6060758

[lipd70048-bib-0018] Gabrielli, F. , L. Golfieri , F. Nascimbeni , P. Andreone , and S. Gitto . 2024. “Metabolic Disorders in Liver Transplant Recipients: The State of the Art.” Journal of Clinical Medicine 13, no. 4: 1014. 10.3390/jcm13041014. 38398327 PMC10889804

[lipd70048-bib-0019] Geer, E. B. , J. Islam , and C. Buettner . 2014. “Mechanisms of Glucocorticoid‐Induced Insulin Resistance: Focus on Adipose Tissue Function and Lipid Metabolism.” Endocrinology and Metabolism Clinics of North America 43, no. 1: 75–102. 10.1016/j.ecl.2013.10.005. 24582093 PMC3942672

[lipd70048-bib-0020] Heit, J. J. , A. A. Apelqvist , X. Gu , et al. 2006. “Calcineurin/NFAT Signalling Regulates Pancreatic Beta‐Cell Growth and Function.” Nature 443, no. 7109: 345–349. 10.1038/nature05097. 16988714

[lipd70048-bib-0021] Huffman, K. M. , D. C. Parker , M. Bhapkar , et al. 2022. “Calorie Restriction Improves Lipid‐Related Emerging Cardiometabolic Risk Factors in Healthy Adults Without Obesity: Distinct Influences of BMI and Sex From CALERIE a Multicentre, Phase 2, Randomised Controlled Trial.” EClinicalMedicine 43: 101261. 10.1016/j.eclinm.2021.101261. 35028547 PMC8741476

[lipd70048-bib-0022] Inker, L. A. , N. D. Eneanya , J. Coresh , et al. 2021. “New Creatinine‐ and Cystatin C–Based Equations to Estimate GFR Without Race.” New England Journal of Medicine 385, no. 19: 1737–1749. 10.1056/NEJMoa2102953. 34554658 PMC8822996

[lipd70048-bib-0023] Kallwitz, E. R. 2015. “Sarcopenia and Liver Transplant: The Relevance of Too Little Muscle Mass.” World Journal of Gastroenterology 21, no. 39: 10982–10993. 10.3748/wjg.v21.i39.10982. 26494955 PMC4607898

[lipd70048-bib-0024] Kuo, T. , A., McQueen , T.‐C., Chen , and J.‐C., Wang . 2015. “Regulation of Glucose Homeostasis by Glucocorticoids.” Advances in Experimental Medicine and Biology 872: 99–126. 10.1007/978-1-4939-2895-8_5. 26215992 PMC6185996

[lipd70048-bib-0025] Kurniawan, L. B. 2024. “Triglyceride‐Glucose Index as A Biomarker of Insulin Resistance, Diabetes Mellitus, Metabolic Syndrome, and Cardiovascular Disease: A Review.” EJIFCC 35, no. 1: 44–51. 38706737 PMC11063788

[lipd70048-bib-0026] Lawendy, B. , S. Srinathan , S. Kotha , et al. 2021. “Systematic Review and Meta‐Analysis of Post‐Transplant Diabetes Mellitus in Liver Transplant Recipients.” Clinical Transplantation 35, no. 7: e14340. 10.1111/ctr.14340. 34033142

[lipd70048-bib-0027] Lee, H. J. , Y. H. Lee , J. S. Kim , et al. 2024a. “#1992 Triglyceride‐Glucose Index and Risk of Cardiovascular Events, Graft Loss and New Onset Diabetes After Transplantation in Renal Transplant Recipients.” Nephrology, Dialysis, Transplantation 39, no. Supplement_1: gfae069‐1015–1992. 10.1093/ndt/gfae069.1015.

[lipd70048-bib-0028] Lee, S. , M. Lee , Y.‐E. Kim , et al. 2024b. “Association of Muscle Mass Loss With Diabetes Development in Liver Transplantation Recipients.” Diabetes & Metabolism Journal 48, no. 1: 146–156. 10.4093/dmj.2022.0100. 38173368 PMC10850281

[lipd70048-bib-0030] Li, D.‐W. , T.‐F. Lu , X.‐W. Hua , et al. 2015. “Risk Factors for New Onset Diabetes Mellitus After Liver Transplantation: A Meta‐Analysis.” World Journal of Gastroenterology 21, no. 20: 6329–6340. 10.3748/wjg.v21.i20.6329. 26034369 PMC4445111

[lipd70048-bib-0029] Li, J.‐X. , and C. L. Cummins . 2022. “Fresh Insights Into Glucocorticoid‐Induced Diabetes Mellitus and New Therapeutic Directions.” Nature Reviews. Endocrinology 18, no. 9: 540–557. 10.1038/s41574-022-00683-6. PMC9116713 35585199

[lipd70048-bib-0031] Liang, X. , K. Lai , X. Li , Y. Li , Z. Xing , and S. Gui . 2025. “Non‐Linear Relationship Between Triglyceride Glucose Index and New‐Onset Diabetes Among Individuals With Non‐Alcoholic Fatty Liver Disease: A Cohort Study.” Lipids in Health and Disease 24, no. 1: 94. 10.1186/s12944-025-02518-5. 40089802 PMC11910846

[lipd70048-bib-0032] Lieber, S. R. , R.‐A. Lee , Y. Jiang , et al. 2019. “The Impact of Post‐Transplant Diabetes Mellitus on Liver Transplant Outcomes.” Clinical Transplantation 33, no. 6: e13554. 10.1111/ctr.13554. 30927288 PMC6995642

[lipd70048-bib-0033] Loosen, S. H. , S. Krieg , S. Chaudhari , et al. 2023. “Prediction of New‐Onset Diabetes Mellitus Within 12 Months After Liver Transplantation—A Machine Learning Approach.” Journal of Clinical Medicine 12, no. 14: 14. 10.3390/jcm12144877. PMC10381881 37510992

[lipd70048-bib-0034] Lv, C. , Y. Zhang , X. Chen , et al. 2015. “New‐Onset Diabetes After Liver Transplantation and Its Impact on Complications and Patient Survival.” Journal of Diabetes 7, no. 6: 881–890. 10.1111/1753-0407.12275. 25676209

[lipd70048-bib-0035] Moon, J. I. , R. Barbeito , R. N. Faradji , J. J. Gaynor , and A. G. Tzakis . 2006. “Negative Impact of New‐Onset Diabetes Mellitus on Patient and Graft Survival After Liver Transplantation: Long‐Term Follow Up.” Transplantation 82, no. 12: 1625–1628. 10.1097/01.tp.0000250361.60415.96. 17198248

[lipd70048-bib-0036] Mulder, F. V. M. , E. F. H. I. Peeters , J. Westerink , F. J. T. Zwartkruis , and W. L. de Ranitz‐Greven . 2022. “The Long‐Term Effect of mTOR Inhibition on Lipid and Glucose Metabolism in Tuberous Sclerosis Complex: Data From the Dutch TSC Registry.” Orphanet Journal of Rare Diseases 17, no. 1: 252. 10.1186/s13023-022-02385-8. 35804402 PMC9264703

[lipd70048-bib-0037] Nayak, S. S. , D. Kuriyakose , L. D. Polisetty , et al. 2024. “Diagnostic and Prognostic Value of Triglyceride Glucose Index: A Comprehensive Evaluation of Meta‐Analysis.” Cardiovascular Diabetology 23, no. 1: 310. 10.1186/s12933-024-02392-y. 39180024 PMC11344391

[lipd70048-bib-0038] Oetjen, E. , D. Baun , S. Beimesche , et al. 2003. “Inhibition of Human Insulin Gene Transcription by the Immunosuppressive Drugs Cyclosporin A and Tacrolimus in Primary, Mature Islets of Transgenic Mice.” Molecular Pharmacology 63, no. 6: 1289–1295. 10.1124/mol.63.6.1289. 12761338

[lipd70048-bib-0039] Peláez‐Jaramillo, M. J. , A. A. Cárdenas‐Mojica , P. V. Gaete , and C. O. Mendivil . 2018. “Post‐Liver Transplantation Diabetes Mellitus: A Review of Relevance and Approach to Treatment.” Diabetes Therapy 9, no. 2: 521–543. 10.1007/s13300-018-0374-8. 29411291 PMC6104273

[lipd70048-bib-0040] Peled, Y. , E. Ram , R. Klempfner , J. Lavee , and E. Raanani . 2021. “Predictive Value of Triglyceride Glucose Index for the Development of New‐Onset Diabetes Mellitus Following Heart Transplantation.” Journal of Heart and Lung Transplantation 40, no. 4: S47–S48. 10.1016/j.healun.2021.01.1855.

[lipd70048-bib-0041] Pereira, M. J. , J. Palming , M. Rizell , et al. 2014. “Cyclosporine A and Tacrolimus Reduce the Amount of GLUT4 at the Cell Surface in Human Adipocytes: Increased Endocytosis as a Potential Mechanism for the Diabetogenic Effects of Immunosuppressive Agents.” Journal of Clinical Endocrinology and Metabolism 99, no. 10: E1885–E1894. 10.1210/jc.2014-1266. 25004245

[lipd70048-bib-0042] Pham, P.‐T. T. , P.‐M. T. Pham , S. V. Pham , P.‐A. T. Pham , and P.‐C. T. Pham . 2011. “New Onset Diabetes After Transplantation (NODAT): An Overview.” Diabetes, Metabolic Syndrome and Obesity: Targets and Therapy 4: 175–186. 10.2147/DMSO.S19027. 21760734 PMC3131798

[lipd70048-bib-0043] Rahimi, L. , A. Rajpal , and F. Ismail‐Beigi . 2020. “Glucocorticoid‐Induced Fatty Liver Disease.” Diabetes, Metabolic Syndrome and Obesity: Targets and Therapy 13: 1133–1145. 10.2147/DMSO.S247379. 32368109 PMC7171875

[lipd70048-bib-0044] Schoening, W. N. , N. Buescher , S. Rademacher , et al. 2013. “Twenty‐Year Longitudinal Follow‐Up After Orthotopic Liver Transplantation: A Single‐Center Experience of 313 Consecutive Cases.” American Journal of Transplantation 13, no. 9: 2384–2394. 10.1111/ajt.12384. 23915357

[lipd70048-bib-0045] Shimada, S. , K. Miyake , D. Venkat , et al. 2024. “Clinical Characteristics of New‐Onset Diabetes After Liver Transplantation and Outcomes.” Annals of Gastroenterological Surgery 8, no. 3: 383–393. 10.1002/ags3.12775. 38707230 PMC11066488

[lipd70048-bib-0047] Sokooti, S. , J. L. Flores‐Guerrero , H. J. L. Heerspink , M. A. Connelly , S. J. L. Bakker , and R. P. F. Dullaart . 2021a. “Triglyceride‐Rich Lipoprotein and LDL Particle Subfractions and Their Association With Incident Type 2 Diabetes: The PREVEND Study.” Cardiovascular Diabetology 20, no. 1: 156. 10.1186/s12933-021-01348-w. 34321006 PMC8320057

[lipd70048-bib-0048] Sokooti, S. , J. L. Flores‐Guerrero , L. M. Kieneker , et al. 2021b. “HDL Particle Subspecies and Their Association With Incident Type 2 Diabetes: The PREVEND Study.” Journal of Clinical Endocrinology and Metabolism 106, no. 6: 1761–1772. 10.1210/clinem/dgab075. 33567068 PMC8118359

[lipd70048-bib-0049] Sokooti, S. , T. Szili‐Török , H. J. L. Heerspink , R. P. F. Dullaart , and S. J. L. Bakker . 2023. “Indirect Insulin Resistance Indices and Their Cut‐Off Values for the Prediction of Post‐Transplantation Diabetes Mellitus in Kidney Transplant Recipients.” Journal of Clinical Medicine 12, no. 23: 7296. 10.3390/jcm12237296. 38068348 PMC10707270

[lipd70048-bib-0050] Soleimanpour, S. A. , M. F. Crutchlow , A. M. Ferrari , et al. 2010. “Calcineurin Signaling Regulates Human Islet {Beta}‐Cell Survival.” Journal of Biological Chemistry 285, no. 51: 40050–40059. 10.1074/jbc.M110.154955. 20943662 PMC3000987

[lipd70048-bib-0051] Song, J.‐L. , W. Gao , Y. Zhong , et al. 2016. “Minimizing Tacrolimus Decreases the Risk of New‐Onset Diabetes Mellitus After Liver Transplantation.” World Journal of Gastroenterology 22, no. 6: 2133–2141. 10.3748/wjg.v22.i6.2133. 26877618 PMC4726686

[lipd70048-bib-0052] Sun, J. , Y. He , L. Bai , et al. 2021. “An Analysis of the Risk Factors for New‐Onset Diabetes Mellitus After Liver Transplantation.” International Journal of General Medicine 14: 4783–4792. 10.2147/IJGM.S324462. 34466023 PMC8402980

[lipd70048-bib-0053] Vaughn, V. M. , D. C. Cron , M. N. Terjimanian , et al. 2015. “Analytic Morphomics Identifies Predictors of New‐Onset Diabetes After Liver Transplantation.” Clinical Transplantation 29, no. 5: 458–464. 10.1111/ctr.12537. 25740081

[lipd70048-bib-0054] Wallia, A. , V. Illuri , and M. E. Molitch . 2016. “Diabetes Care After Transplant: Definitions, Risk Factors, and Clinical Management.” Medical Clinics of North America 100, no. 3: 535–550. 10.1016/j.mcna.2016.01.005. 27095644

[lipd70048-bib-0055] Younossi, Z. , M. Stepanova , S. Saab , G. Trimble , A. Mishra , and L. Henry . 2015. “The Association of Hepatitis C Virus Infection and Post‐Liver Transplant Diabetes: Data From 17 000 HCV‐Infected Transplant Recipients.” Alimentary Pharmacology & Therapeutics 41, no. 2: 209–217. 10.1111/apt.13027. 25413020

[lipd70048-bib-0056] Zhang, Z. , J. Sun , M. Guo , and X. Yuan . 2023. “Progress of New‐Onset Diabetes After Liver and Kidney Transplantation.” Frontiers in Endocrinology 14: 1091843. 10.3389/fendo.2023.1091843. 36843576 PMC9944581

[lipd70048-bib-0057] Zhong, H. , L. Luo , X. Wang , and Y. Xiao . 2025. “Association Between Triglyceride to HDL Cholesterol Ratio and a Risk of Diabetes Mellitus: A Systematic Review and Meta‐Analysis.” Laboratory Medicine 56, no. 1: 1–6. 10.1093/labmed/lmae052. 39066659

