# Supplementary material for: Triacylglycerol‐Based Insulin Resistance Indices and Post‐Transplantation Diabetes Mellitus After Liver Transplantation
Source: Lipids. 2026 Mar 6;61(4):479–92. doi: 10.1002/lipd.70048 (PMC13341382; doi:10.1002/lipd.70048)

**Supplementary Materials**

**Table S1: Baseline characteristics of participants by cohort after propensity score matching**

|  | **No T2D at Baseline** | | | **T2D at Baseline** | | |  | |
| --- | --- | --- | --- | --- | --- | --- | --- | --- |
| **Variable** | **LTR (n = 246)** | **PREVEND (n = 246)** | **p¹** | **LTR (n = 121)** | **PREVEND (n = 121)** | **p²** | **p^3^** | **p^4^** |
| Age (years) | 52.6 (15.0) | 53.7 (13.8) | 0.400 | 61.1 (11.2) | 62.9 (10.4) | 0.182 | <0.001 | <0.001 |
| Sex, % |  |  | 0.526 |  |  | 0.682 | 0.009 | 0.028 |
| Male | 53.7 | 56.9 |  | 68.6 | 65.3 |  |  |  |
| Female | 46.3 | 43.1 |  | 31.4 | 34.7 |  |  |  |
| BMI (kg/m²) | 25.8 (4.5) | 25.7 (3.6) | 0.785 | 28.1 (5.3) | 28.9 (4.6) | 0.199 | <0.001 | <0.001 |
| Smoking, % |  |  | 0.491 |  |  | 0.314 | 0.089 | 0.304 |
| No | 89.0 | 86.6 |  | 95.0 | 90.9 |  |  |  |
| Yes | 11.0 | 13.4 |  | 5.0 | 9.1 |  |  |  |
| Alcohol, % |  |  | 0.909 |  |  | 0.565 | 0.007 | 0.017 |
| 0 | 67.9 | 69.1 |  | 82.6 | 76.9 |  |  |  |
| 0.1–10 | 20.7 | 19.1 |  | 13.2 | 18.2 |  |  |  |
| 10–30 | 9.3 | 8.9 |  | 1.7 | 0.8 |  |  |  |
| > 30 | 2.0 | 2.8 |  | 2.5 | 4.1 |  |  |  |
| History CVD, % |  |  | 0.213 |  |  | 0.465 | 0.028 | 0.020 |
| No | 94.7 | 91.5 |  | 87.6 | 83.5 |  |  |  |
| Yes | 5.3 | 8.5 |  | 12.4 | 16.5 |  |  |  |
| HTN med, % |  |  | 0.010 |  |  | 0.001 | 0.001 | 0.236 |
| No | 60.6 | 72.0 |  | 42.1 | 63.6 |  |  |  |
| Yes | 39.4 | 28.0 |  | 57.9 | 36.4 |  |  |  |
| SBP (mm Hg) | 131.1 (16.7) | 131.9 (21.3) | 0.658 | 134.3 (16.5) | 135.4 (20.4) | 0.642 | 0.089 | 0.098 |
| DBP (mm Hg) | 80.4 (10.7) | 75.6 (9.4) | <0.001 | 78.1 (11.4) | 76.3 (9.5) | 0.197 | 0.042 | 0.527 |
| eGFR (mL/min/1.73 m²) | 82.2 (24.6) | 82.2 (22.3) | 0.993 | 70.4 (24.7) | 79.8 (21.8) | 0.002 | <0.001 | 0.227 |
| Glucose (mg/dL) | 95.0 (17.0) | 84.9 (10.8) | <0.001 | 141.6 (52.2) | 144.3 (44.4) | 0.665 | <0.001 | <0.001 |
| Total chol. (mmol/L) | 4.4 (1.1) | 5.4 (1.0) | <0.001 | 4.2 (0.9) | 5.2 (1.1) | <0.001 | 0.061 | 0.199 |
| HDL-c (mmol/L) | 1.5 (0.42) | 1.3 (0.30) | <0.001 | 1.27 (0.46) | 1.04 (0.23) | <0.001 | <0.001 | <0.001 |
| LDL-c (mmol/L) | 2.3 (0.79) | 3.5 (0.85) | <0.001 | 2.0 (0.63) | 3.4 (0.99) | <0.001 | 0.002 | 0.428 |
| Triacylglycerol (mmol/L) | 1.4 (0.68) | 1.4 (1.1) | 0.778 | 1.9 (1.0) | 1.8 (1.0) | 0.144 | <0.001 | 0.002 |
| TyG index | 8.5 [8.1–8.8] | 8.3 [8.0–8.7] | 0.001 | 9.2 [8.8–9.6] | 9.2 [8.8- 9.5] | 0.653 | <0.001 | <0.001 |
| TAG/HDL‑C ratio | 0.84 [0.57–1.33] | 0.89 [0.61–1.47] | 0.210 | 1.55 [0.87–2.14] | 1.43 [1.05–2.16] | 0.652 | <0.001 | <0.001 |

**Abbreviations**: T2D, type 2 diabetes mellitus; LTR, liver transplant recipients; PREVEND, Prevention of REnal and Vascular ENd‐stage Disease study; BMI, body mass index; CVD, cardiovascular disease; HTN med, antihypertensive medication; SBP, systolic blood pressure; DBP, diastolic blood pressure; eGFR, estimated glomerular filtration rate; Glucose, fasting plasma glucose (mg/dL); TC, total cholesterol; HDL-c, high‑density lipoprotein cholesterol; LDL-c, low‑density lipoprotein cholesterol; TAG, triacylglycerol; TyG index, triacylglycerol ‑glucose index [ln(TAG × Glucose/2)]; TAG/HDL‑c ratio, log‑transformed triacylglycerol ‑to‑HDL‑cholesterol ratio [ln(TAG/HDL-c)]. Data are shown as mean ± SD for normally distributed variables, median [IQR] for non‑normal variables, and percentages for categorical variables. p¹ denotes the p‑value for the comparison of LTR vs PREVEND without T2D at baseline; p^2^ denotes the p‑value for the comparison of LTR vs PREVEND with T2D at baseline. p^3^ denotes the comparison of LTR with and without T2D at baseline; p^4^ denotes the comparison of PREVEND with and without T2D at baseline.

**Figure S1: Flow-chart of Participant Distribution**


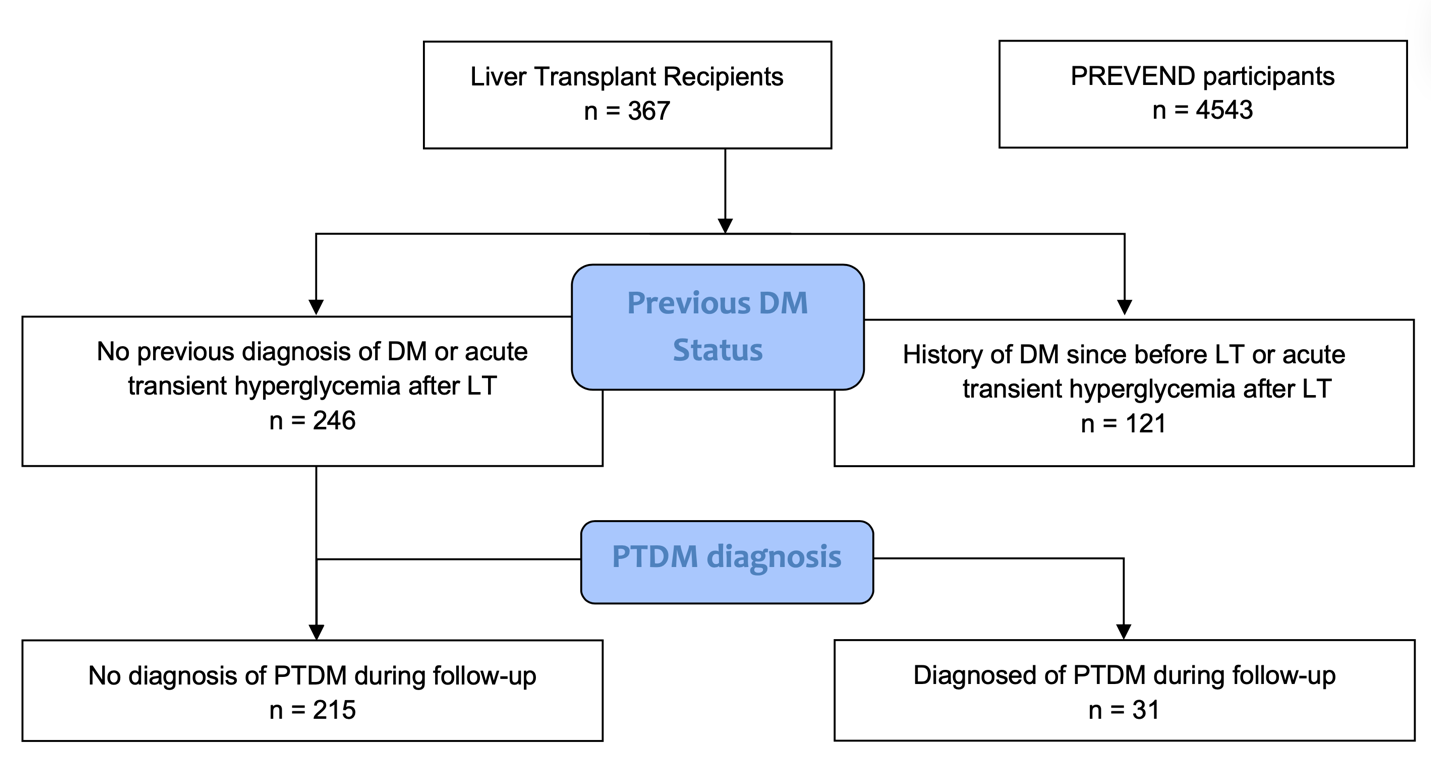


DM: diabetes mellitus, PREVEND: Prevention of Renal and Vascular End-Stage Disease, PTDM: post-transplant diabetes mellitus

**Figure S2: Forest Plot of the Stratified Analysis Showing Association Between TyG and TAG/HDL-c, with the Risk of Incident Diabetes in Cox regression and Logistic regression Models**


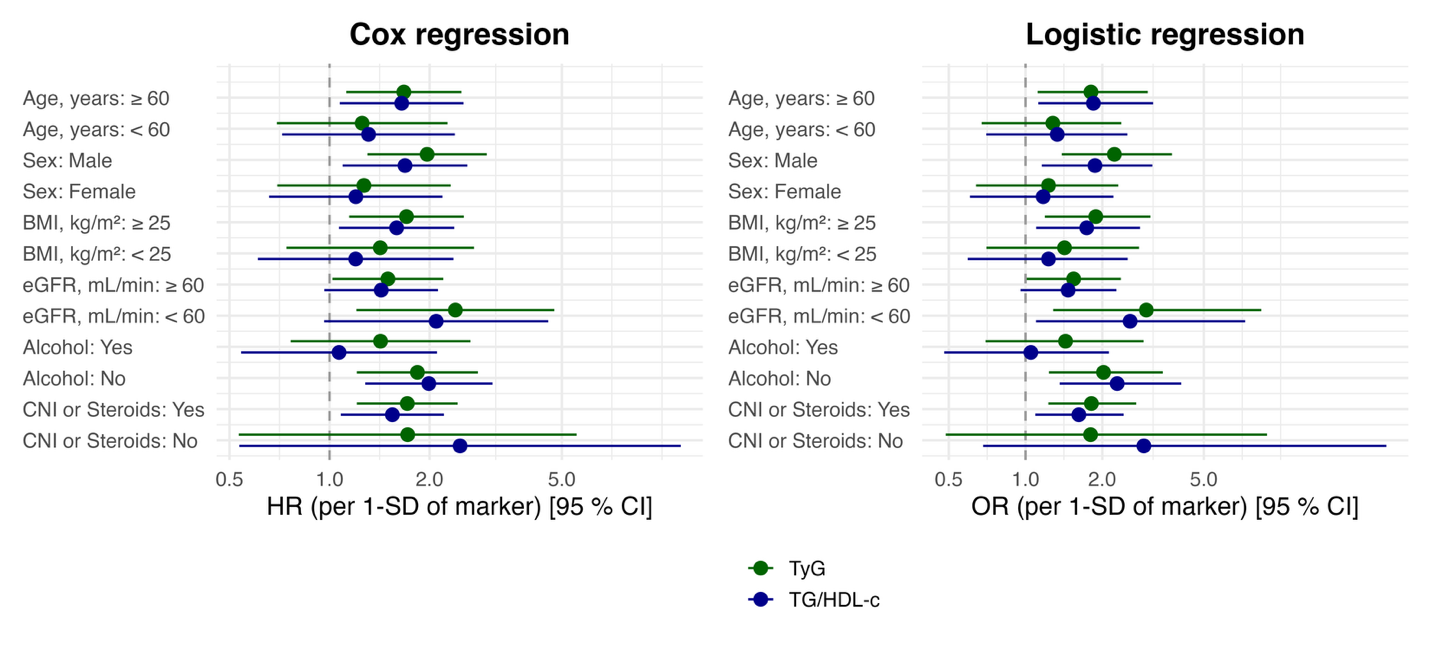

Supplement: Supplementary file 1 — Data S1: lipd70048‐sup‐0001‐Supinfo.docx. [file LIPD-61-479-s001.docx]
